# Enzymatic and non-enzymatic comparison of two different industrial tomato (*Solanum lycopersicum*) varieties against drought stress

**DOI:** 10.1186/s40529-017-0186-6

**Published:** 2017-08-02

**Authors:** Özge Çelik, Alp Ayan, Çimen Atak

**Affiliations:** Department of Molecular Biology and Genetics, Faculty of Science and Letters, Istanbul Kultur Univesity, Ataköy, 34156 Istanbul, Turkey

**Keywords:** *Solanum lycopersicum*, Drought stress, Antioxidant enzyme activities, Isozyme pattern

## Abstract

**Background:**

The aim of this study is to compare the tolerance mechanisms of two industrial tomato varieties (X5671R and 5MX12956) under drought stress. 14 days-old tomato seedlings were subjected to 7 days-long drought stress by withholding irrigation. The effects of stress were determined by enzymatic and non-enzymatic parameters. The physiological damages were evaluated via lipid peroxidation ratio, total protein content, relative water content, chlorophyll content and proline accumulation. Enzymatic responses were determined by biochemical analysis and electrophoresis of SOD, APX, POX and CAT enzymes.

**Results:**

Relative water contents of X5671R and 5MX12956 varieties at 7th day of drought were decreased to 8.4 and 12.2%, respectively. Applied drought decreased all photosynthetic pigments of X5671R and 5MX12956 varieties during the treatment period significantly comparing to the Day 0 as the control. Total protein content, lipid peroxidation and proline accumulation presented increased values in both varieties in accordance with the increasing stress intensity. According to lipid peroxidation analysis, 5MX12956 tomato variety was found more drought sensitive than X5671R variety. Antioxidative enzyme activities showed increases in both varieties as a response to drought stress, although CAT and APX activities presented decrease on the 7th day of applied stress. 7 days long drought stress differentially altered POX, APX and SOD isozyme patterns. Same POX bands were observed in both varieties with different band intensities.

**Conclusions:**

However, main isozyme pattern differences were obtained for SOD and APX. APX1, Fe-SOD and Cu/Zn-SOD2 isozyme bands should be evaluated to define their main role in the tolerance mechanism of both tomato varieties.

## Background

Drought stress is described as one of the most harmful natural hazards that limits the growth, productivity and crop yield of plants. The aggressive usage of natural resources and increase in the global climate temperatures are two main factors causing drought. The general effects of drought stress have been studied in different plant species. The main inhibitory features of drought stress were observed on cell proliferation and expansion, leaf size, stem elongation, root proliferation alongside with crop growth and biomass accumulation (Harrison et al. 2014; Sekmen et al. 2014; Shanker et al. 2014; Zdravkovic et al. 2013). On the other hand, plants involve specific protective adaptation mechanisms against short term and long term drought. Plants can either enhance response mechanisms to avoid the effects of drought or tolerate the adverse effects of the stress. Plants can reduce leaf size and number, increase the area and length of roots to absorb more water, produce thicker cuticula and wax layers, control stomatal closure rates, induce cell turgor maintenance mechanisms such as accumulation of compatible solutes, reduce light absorbance by reflecting the exposed sun light to reduce the water loss by transpiration and the alternative mechanisms to reduce toxic oxidative radicals which were produced by the stress (Harb et al. 2010; Shanker et al. 2014). Therefore, they can avoid the short term drought until the life cycle is completed to produce next the generation. Short term drought stress depends on random climate changes and is mostly predictable. Long term drought stress depends on more complex weather features and needs advanced molecular mechanisms to enhance increased transcription rates of functional and regulatory genes involved in drought tolerance (Harb et al. 2010; Jeanneau et al. 2002; Shanker et al. 2014). In next decades, the drought effected area is expected to enlarge around the world following the reducing sustainable water resources and increasing long term water deficiency situations. Comprehensive physiological and molecular studies are necessary for both in vitro and field conditions to evaluate existing crop varieties and improving them.

Tomato (*Solanum lycopersicum*) which is a member of Solanaceae family, is one of the most important agricultural plant worldwide, because of its industrial products and nutritional content (George et al. 2013; Zdravkovic et al. 2013). According to the 2012 world tomato production report of FAO, Turkey was in fourth place by 11,350,000 million tons of production following China (50,000,000), India (17,500,000) and United States (13,206,950) (FAO 2012). Tomato is a sensitive plant against various environmental abiotic stress factors. It is known fact that heat and drought stresses have the most limiting effects on tomato varieties among all other abiotic stress factors. Especially, drought stress during vegetative and early reproductive periods of tomato life cycle reduces yield dramatically (George et al. 2013; Wahb-Allah et al. 2011; Zdravkovic et al. 2013).

As the other abiotic stress factors, drought causes imbalance on equilibrium between reactive oxygen species (ROS) such as hydroxyl radicals (OH^**·**^), hydrogen peroxide (H_2_O_2_), singlet oxygen (^1^O_2_), superoxide radical (^−^O_2_) and scavenging activities of antioxidant system elements. Reactive oxygen species are products of metabolic processes taking place in different compartments of cell such as chloroplasts, mitochondria and peroxisomes. ROS, which are the group of free radicals derived from O_2_, are consisted of reactive molecules and ions (Gill and Tuteja 2010). These free radicals which have high reaction potential are harmless at ground levels and have lifetime in unit of micro seconds until they are scavenged in cell. This short lifetime makes ROS effective only in several 100 nm radius. However, when cellular components undergo to oxidative stress, biomolecules as proteins, unsaturated fatty acids, enzymes and nucleic acids are exposed to damaging effects of ROS. Loss or malfunction of key biomolecules may even trigger death of cells depending on the duration and intensity of stress. The relation between the antioxidant system elements to suppress toxic levels of ROS within the cell, is physiologically balanced. Therefore, plants initiate enzymatic (POX, EC1.11.1.7; APX, EC1.11.1.1; SOD, EC1.15.1.1; CAT, EC1.11.1.6) and non-enzymatic (carotenoids, ascorbate, glutathione, tocopherols) defense mechanisms to obtain oxidative homeostasis (Bartosz 1997; Mittler 2002; Sekmen et al. 2014). As known from previous reports, different plant varieties may differ on metabolic pathways. Biochemical strategies are getting important to enhance tolerance capacities of plants under drought conditions. Induction of antioxidant enzyme activities is known to have the major role in survival against drought. As a result, it is getting important to detect changes on enzymatic and non-enzymatic antioxidant systems to reveal metabolic pathways of drought stress tolerance of different plant varieties. Therefore, target oriented breeding and selection studies can be conducted to improve economically important crop plants such as tomato against several environmental abiotic stress factors as drought.

In the present study, antioxidant systems of two economically important industrial tomato varieties (X5671R and 5MX12956) were investigated against drought stress. Physiological and biochemical characteristics of these two varieties were evaluated under intensive drought conditions according to the activities of antioxidants as POX, APX, CAT, SOD and their isozyme profiles. Both varieties were evaluated for their isozyme band diversities to identify their tolerance responses against drought.

## Methods

### Plant material

X5671R and 5MX12956 industrial tomato seeds were provided from Agromar Brand Seeds Company in Bursa/Turkey. Seeds were germinated in 252-well styrofoam filled with perlite and watered by Scotts fertilizer (1.2 µS EC) for 14 days. Drought stress was applied by withholding watering starting from 14th day for all experimental groups, except the control group (Day 0). Samples of both varieties were collected daily during 7 days period and stored at −20 °C for further analysis. All treatments were designed as three independent replicates and each treatment contained 36 plantlets.

### Relative water content

Three random leaves from the same part of each treatment group were collected to determine relative water contents. After measuring the fresh weights (FW), leaves were placed into the distilled water for 8 h in order to obtain turgid weight. Following the turgid weight (TW) measurement, leaves were heated in dry heat incubator for 24 h to obtain dry weights. Relative water contents (RWC) were calculated according to the formula as reported by Smart (1974):

$${\text{RWC}} \;( \%) = \frac{{{\text{FW}} - {\text{DW}}}}{{{\text{TW}} - {\text{DW}}}} \times 100$$

### Chlorophyll and carotenoid content

Chlorophyll a, chlorophyll b, total chlorophyll and carotenoid contents of X5671R and 5MX12956 tomato varieties were calculated according to the spectrophotometric method described by Arnon (1949). Leaf tissues were grinded by cold %80 acetone and homogenate filtered through Whatman No:1 filter paper. Absorbance values were measured at 663 and 645 nm wavelengths by using acetone as blank.

### Lipid peroxidation

Drought stress is known to induce lipid peroxidation which indicates the level of oxidative damage on cellular membranes. MDA which is a decomposition product of lipid peroxidation was measured according to method of Stewart and Bewley (1980) for all experimental groups. MDA concentrations were calculated by using an extinction coefficient of 155 mM^−1^ cm^−1^. The results were expressed as μmol MDA g^−1^ FW.

### Proline levels

Proline levels were determined according to the method of Bates et al. (1973). Extracts were prepared in 3% sulphosalicylic acid. Filtered extracts were boiled at 100 °C for 1 h following the addition of acid ninhydrin reagent (ninhydrin and glacial acetic acid). Reaction was stopped in ice bath and fractions were separated by using toluene. Absorbance values were measured at 520 nm wavelength. Proline levels were calculated by using standard calibration curve in unit of µg µL^−1^ proline g^−1^ FW.

### Total protein extraction and enzyme activity assays

Total protein extractions for all enzymatic analysis (activity and isozyme assays) were performed by using the same buffer except ascorbate peroxidase (APX) assays. 0.5 g of tomato leaves were grounded by liquid nitrogen and homogenized in 1.25 mL of 50 mM Tris–HCl (pH 7.8) containing 0.1 mM EDTA, 0.2% Triton X-100, 1 mM PMSF and 2 mM DTT. For APX enzyme extraction, 5 mM sodium ascorbate was added to the buffer and DTT was replaced by 2% PVP. All extractions were performed at 4 °C. Samples were centrifugated at 14,000 rpm for 30 min after the extraction. Supernatants were stored at 4 °C until the analysis. Total protein contents were calculated by using bovine serum albumin as protein standard according to the method described by Bradford (1976) and described as µg g^−1^ FW.

The soluble protein profiles were visualized by SDS-PAGE. 75 μg of each sample were loaded to the 4% stacking gel and 12% separating gels according to the method of Laemmli (1970). Electrophoresis was continued at 30 mA constant current until the tracking dye reached to the bottom of the gel. Separate native-PAGE gels were used to identify the isozymes for each enzyme.

POX activity (EC1.11.1.7) was calculated by the increase in absorbance at 470 nm followed for 90 s according to the method of Seevers et al. (1971). The reaction mixture of 0.1 M phosphate buffer (pH 7.0) contained 20 mM guaiacol and 20 mM H_2_O_2_. A unit of POX activity was defined as mmol H_2_O_2_ decomposed ml^−1^ min^−1^.

APX (EC1.11.1.1) was measured due to decrease in absorbance at 290 nm as a result of the oxidization of ascorbate according to the method of Nakano and Asada (1981). The reaction mixture contained 50 mM Na-phosphate buffer (pH 7.0) including 50 mM ascorbate, 0.1 mM Na_2_-EDTA, 1.2 mM H_2_O_2_. APX was calculated by using the coefficient of 2.8 mM^−1^ cm^−1^. One unit of APX was defined as 1 mmol ml^−1^ ascorbate oxidized min^−1^.

SOD (EC1.15.1.1) activity was assayed according to the method of Beauchamp and Fridovich (1971) based on the photochemical reduction ability of NBT by SOD. Assays were carried out at 25 °C by using the reaction buffer of 50 mM Na-phosphate buffer (pH 7.8) including 0.033 mM NBT, 10 mM l-methionine, 0.66 mM Na_2_EDTA and 0.0033 mM riboflavin. Reactions were initiated by addition of riboflavine to the mixture at last. After 10 min of incubation under intense light, the absorbance values were determined at 560 nm. SOD activity was defined as % inhibition of NBT by photoreduction activity of the enzyme.

CAT (EC1.11.1.6) activity was determined by the method of Aebi (1984) due to the disappearance of H_2_O_2_ at 240 nm. Reaction mixture contained 50 mM Na-phosphate buffer (pH 7.0) including 30 mM H_2_O_2_. The decrease in absorbance was followed for 90 s. 1 mmol H_2_O_2_ ml^−1^ min^−1^ was defined as 1 U of CAT activity.

### Identification of isozymes

Electrophoretic separations were performed by using 4% stacking and 12% separating native polyacrylamide gels under constant current of 120 mA at 4 °C. To determine the SOD isozymes, equal amount of protein samples (75 µg/well) were loaded as described by Laemmli (1970). After electrophoretic separation, SOD isozymes were identified according to the photochemical staining method described by Beauchamp and Fridovich (1973). SOD isozymes were differentiated by using SOD inhibitors. The selective inhibitions were performed by 2 mM KCN for Cu/Zn-SOD activity and 3 mM H_2_O_2_ for Cu/Zn-SOD and Fe-SOD. Mn-SOD is resistant to both inhibitors.

POX isozymes were detected by the method of Seevers et al. (1971). 50 µg/well protein extracts were loaded. Gels were stained by 0.2 M Na-phosphate buffer (pH 7.0) containing 20 mM H_2_O_2_ and 20 mM guaiacol after separation. Reaction was stopped by incubating the gels in 7% acetic acid.

APX gel staining was performed according to the method described by Mittler and Zilinskas (1991). Method is based on inhibition of NBT by ascorbate. Gels were pre-run without loading samples to gel in running buffer (pH 7.0) containing 2 mM sodium ascorbate for an hour to equilibrate gel before the electrophoretic separation. After the separation, gels were incubated in 50 mM potassium phosphate buffer (pH 7.0) containing 2 mM sodium ascorbate for 30 min, then transferred to potassium phosphate buffer (pH 7.8) containing 4 mM sodium ascorbate and 2 mM H_2_O_2_ for 20 min. Finally, gels were stained by 50 mM potassium phosphate buffer (pH 7.8) containing 28 mM TEMED and 2.5 mM NBT for 20 min.

The isoenzyme bands were named in the order to their migration distances. Gels were visualized by Bio-RAD Doc XR Image system. Band intensity profiles were quantified by GelQuant.NET version 1.8.2 software.

### Statistical analysis

The experiments were repeated three times, and each data point was the mean of three replicates. The results were expressed as mean and error bars were used for showing standard error of mean values (±SEM). Statistical evaluation of the means was performed by using one-way ANOVA analysis and statistically meaningful data were compared using Student–Newman Keuls test performed by using GraphPad Prism version 4.00 for Windows statistics software. Different letters in graphs indicates significant differences between treatments (P < 0.05).

## Results and discussion

Drought stress effects various metabolic processes adversely via altering key biomolecules by causing oxidative stress. Different plant species and varieties may involve alternative response strategies against drought stress. Studying on defense mechanisms against drought stress for several plant species might have importance to reveal new breeding strategies (Farooq et al. 2009; Griffiths and Parry 2002). The main aspect of this study is to evaluate enzymatic and non-enzymatic responses of two industrial tomato varieties against 7 days long drought conditions.

### Relative water contents

The inadequate water uptake caused by drought stress leads plants to lose water as a result of metabolic processes depending on subjected stress level. Therefore, plants involve various strategies to conserve cellular water in response to increasing drought intensity. The availability of the controlled mechanism is an important factor to define tolerance of different varieties against drought. In this context, many studies reported alterations on relative water contents of various crop plants depending on the duration of applied drought (Alexieva et al. 2001; Bai et al. 2006; Keyvan 2010; Saeidi and Zabihi-e-Mahmoodabad 2009).

**Fig. 1:**
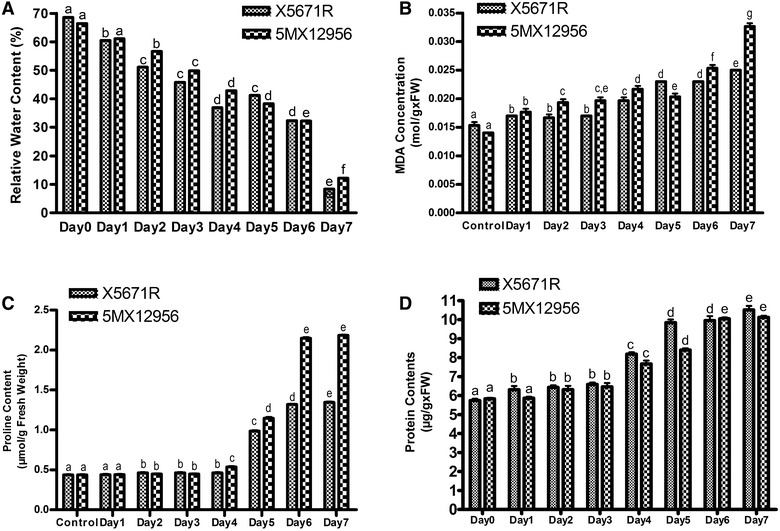
Effects of 7 day drought stress on relative water content (**A**), lipid peroxidation rate (**B**), proline content (**C**) and total protein concentration (**D**) of X5671R and 5MX12956 tomato seedlings. Data represent the average of three independent experiments with three replicates. *Vertical bars* indicate ± SEM and the *differentially given letters* represent significance at the 0.05 level

After 7 days of drought treatment, both varieties lost relative water contents dramatically. RWC of X5671R variety was decreased to the level of 8.4% at 7 day of stress, which was 68.5% before the treatment. 5MX12956 variety presented relatively lower decrease on RWC compared to the X5671R variety. RWC of 5MX71R was decreased to the level of 12.2% at 7th day from the level of 66.4% measured at Day 0 (Fig. 1A).

In the present study, relative water contents (RWC) of the leaves of X5671R and 5MX12956 varieties decreased significantly. Decrease was gradual in response to the increasing drought stress through 7 days period. Saruhan et al. (2010) reported decreases in relative water content in the leaf and petiole during the drought period.

### Lipid peroxidation

Abiotic stress factors as drought cause oxidative stress by disturbing the balance between antioxidants and reactive oxygen species. MDA which is a product of peroxidation of lipid membranes in cells can be used as an indicator of oxidative stress in plants. In our study, lipid peroxidation levels started to increase from 1st day for both varieties. The lipid peroxidation rates were given in Fig. 1B. The lipid peroxidation rates were found 52.34 and 57.28% increased at 7th day of stress as an indicator of oxidative stress in X5671R and 5MX12956 tomato varieties, respectively (Fig. 1B). The regression analysis between the MDA levels of both varieties were found statistically significant (P < 0.05). According to the experimental results of lipid peroxidation, 5MX12956 tomato variety was determined as more drought sensitive than X5671R variety.

MDA concentration was gradually increased and statistical significance was determined from the beginning of the drought treatment with respect to Day 0 for both varieties. According to the results of MDA content, X5671R tomato variety was found as more drought tolerant than 5MX12956 variety. Furthermore, the increase in MDA levels was proportional to the linear decrease in the relative water contents which offers related pattern between increasing oxidative stress and lipid peroxidation.

Sánchez-Rodríguez et al. (2012) applied drought by non-irrigation to 48 day-old tomato plants sown in perlite under greenhouse conditions. They reported MDA increase for unirrigated tomato varieties comparing to the irrigated control group. Also applied drought stress to two different tomato varieties by withholding irrigation on 22 days old plants. They reported significant MDA increase after drought treatment.

### Proline content

Plants gain tolerance by osmolyte accumulation under stress conditions. Proline is one of the compatible solutes that plays roles in plants as osmotic regulator during water deficiency and also takes place in defense systems against other environmental stress factors. Because of its osmotic regulation function during drought, many studies reported drought induced accumulation of proline (Hong-Bo et al. 2006; Mohammadkhani and Heidari 2008a).

In our study, proline levels were increased in both tomato varieties as response to drought stress during 7 days and the values were presented in Fig. 1C. The increase in proline levels was found statistically significant at 2nd day of the stress for 5MX12956 and X5671R varieties, respectively. A sharp increase was observed at the 5th day of stress for both varieties. At the 5th day of stress, the proline level of X5671R was 2.25-fold increased while 2.63-fold increase was observed for 5MX12956 variety in comparison to Day 0 (P < 0.05). Proline levels were 3.08- and 5.01-fold increased after 7 days of drought treatment for X5671R and 5MX12956 tomato varieties, respectively. Mohammadkhani and Heidari (2008a) were also reported a statistically significant proline accumulation after 2nd day of drought application suggesting increase on proline synthesis enzymes and decrease on proline degradation (P < 0.05).

### Soluble protein content

Various alterations occur on the physiological and biochemical pathways of the plants during environmental stresses as drought. Drought stress may effect protein content of the plants differently depending on the duration and the severity of the stress. Mohammadkhani and Heidari (2008b) reported that accumulation of some proteins and enzymes especially related to glycolysis, Krebs cycle and lignin synthesis increased during drought stress. However, total soluble protein content of plants can also be reduced when the level of oxidative stress was higher than the tolerance capacity of defense system of the plants (Celik and Atak 2012).

Changes in total protein content caused by drought stress treatment were given in Fig. 1D. Statistically significant increase in protein contents was measured from the first day of stress for X5671R variety, while the significance was observed from the 2nd day for 5MX12956 variety with respect to Day 0 (P < 0.05). In our present study, linear increase on total protein contents of both varieties in response to increasing drought severity was observed. This result suggest involvement of increased enzymatic and non-enzymatic proteins as a response to drought in both varieties. Several studies showed relationship between accumulation of drought-induced proteins such as dehydrins and dehydrin-like proteins and physiological adaptations in water limitations (Mohammadkhani and Heidari 2008b).

### Chlorophyll and carotenoid contents

**Fig. 2:**
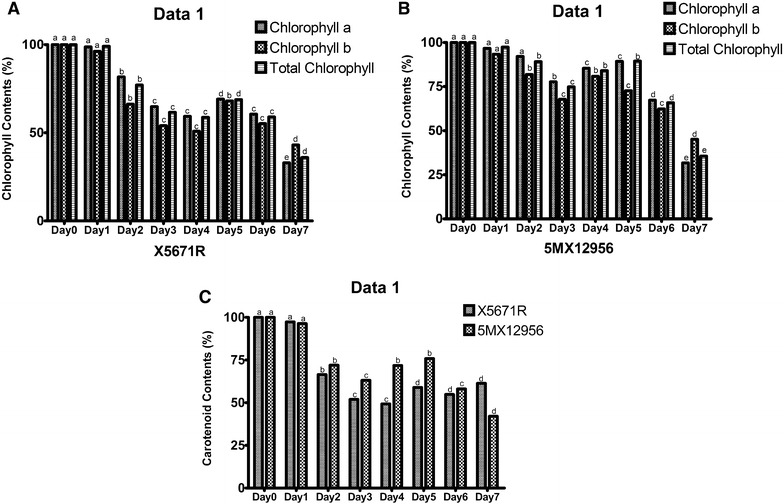
Effects of 7 days-long drought stress on photosynthetic pigment concentrations of X5671R (**A**) and 5MX12956 (**B**) and carotenoid (**C**). In all results, Day 0 is estimated as 100% and calculations presented as percentage changes comparing to the Day 0

Photosynthetic pigments play important roles in harvesting photons for energy metabolism of plants and also have role in stress response against oxidative stress conditions. In many studies chlorophyll and carotenoid contents of different crop plants reported to stay unchanged or decreased depending on the drought application and duration of stress (Farooq et al. 2009). Talebi et al. (2013) reported statistically significant decrease on photosynthetic pigment contents in 35 different Kabuli chickpea varieties which were subjected to drought stress by non-irrigation for 3 weeks. We observed decrease in chlorophyll a, chlorophyll b, total chlorophyll and carotenoid levels of both varieties in a linear manner by increasing drought intensity. The decrease of photosynthetic pigment concentration was one of the signs of reduction in growth and metabolic processes after water loss (Farooq et al. 2009). Drought treatment decreased all photosynthetic pigments of X5671R and 5MX12956 varieties significantly, comparing to the Day 0. Chlorophyll a content decreased to the levels of 33.0 and 31.7% at the 7th day of the drought stress, respectively (Fig. 2A, B). Similar decrease pattern was observed for chlorophyll b and total chlorophyll contents (Fig. 2A, B). Chlorophyll b decreased to the levels of 43.1 and 45.1% while total chlorophyll levels were 36.0 and 35.6%, respectively at the 7th day of the applied drought (P < 0.05). Effects of drought were also observed on carotenoid content. Carotenoid levels decreased in all treatment days and decreased 38.6 and 57.9% at 7th day of the stress respectively, compared to the Day 0 (P < 0.05) (Fig. 2C).

### Enzymatic parameters

Another important stress scavenging mechanism is related to antioxidant enzyme activity profiles during stress. Increased stress tolerance is mostly seen as a result of increased antioxidant enzyme activities. To evaluate the enzymatic responses of X5671R and 5MX12956 tomato varieties, we measured enzymatic activities of SOD, POX, APX and CAT.

#### Antioxidant enzyme activities

**Fig. 3:**
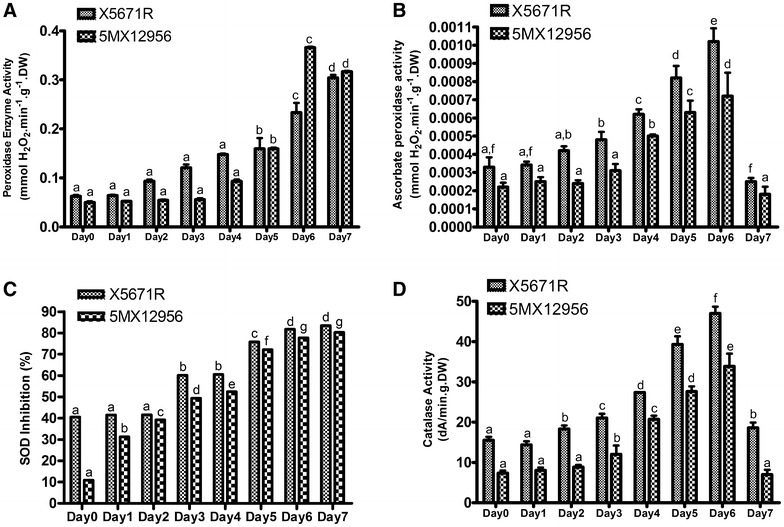
Effects of 7 day drought stress on the activities of POX (**A**), APX (**B**), SOD (**C**) and CAT (**D**) of X5671R and 5MX12956 tomato seedlings. Data represent the average of three independent experiments with three replicates. *Vertical bars* indicate ± SEM and the *differentially given letters* represent significance at the 0.05 level

Peroxidase is an antioxidant enzyme which catalyse H_2_O_2_ dependent oxidation reactions (Acar et al. 2001; Demiral and Turkan 2005). Peroxidase enzyme activities of two varieties subjected to the 7 days of drought stress are presented in Fig. 3A. Peroxidase activities of both varieties presented increasing profile during the 7 days of stress. However, in both varieties, statistically significant POX activity increases were determined on 5th day of the treatment (P < 0.05). The highest activity increase was observed on the 6th day of stress for 5MX12956 variety by 7.4-fold. While this activity decreased to 6.4-fold, X5671R variety presented gradual increase and POX activity reached to 4.76-fold on 7th day of treatment, with respect to the Day 0.

Bai et al. (2006) applied drought stress to maize plants in soil conditions by reducing relative water of soil. They reported increasing peroxidase enzyme activity related to the drought severity. Chakraborty and Pradhan (2012) experimented enzymatic performance of five different wheat varieties in response to 3, 6 and 9 days of drought stress applied by withholding watering in pots. They reported statistically significant increases on peroxidase activities of different wheat varieties depending on the duration of stress. According to our results, while the first statistically significant activity increases presented in 5th day, peroxidase activity was extended until the end of applied stress period for X5671R variety, although 5MX12956 variety presented the highest activity in 6th day and the activity was decreased in 7th day of treatment.

Ascorbate peroxidase enzyme uses ascorbic acid to reduce H_2_O_2_ in plants which is generated in increased levels by many environmental stress factors as drought, salt, high and low temperatures (Caverzan et al. 2012; Shigeoka et al. 2002). As shown in the Fig. 3B, APX enzyme activities were increased due to the increasing drought stress through 6 days. After 6th day, APX enzyme activities of both varieties were decreased below the untreated control group by the increasing oxidative stress. APX activity of 5MX12956 variety was not statistically significant for Day 0 and Day 7 of treatment. At 6th day of stress treatment APX activities of X5671R and 5MX12956 varieties were reached to the maximum levels of 3.1- and 3.3-fold, respectively.

Zlatev et al. (2006) applied drought stress to three different 14-days old bean varieties by withholding irrigation until RWC of leaves reach to 65%. They reported increased APX enzyme activities for all three varieties suggesting APX is involving H_2_O_2_ scavenging in bean. Chugh et al. (2011) reported increased APX activities for drought tolerant maize varieties while sensitive varieties presented decreased APX. These results suggested that increased APX activity might be adaptive response against the drought. Zhang and Kirkham (1996) applied drought stress to sunflower and sorghum seeds in pot conditions by withholding watering. They reported sunflower APX enzyme activity was not effected from applied drought stress, while sorghum plants decreased APX activity greatly. They suggested this may indicate greater accumulation of H_2_O_2_ in cells that can result decreased synthesis of APX and increased degradation of enzyme. In the present study, drought conditions were more intense and RWC of both tomato varieties decreased to the levels below 20%. Both tomato varieties presented increased APX enzyme activity (P < 0.07), however the oxidative stress level was beyond the scavenging capacity of the enzyme at 7th day.

Figure 3C shows the results of SOD enzyme activities of X5671R and 5MX12956 varieties which were subjected to drought stress by non-watering for 7 days. Both varieties increased SOD activities following the increasing oxidative stress induced by the drought conditions. X5671R variety showed no statistically significant changes in enzyme activity until the 3rd day of the treatment, while 5MX12956 variety was effected from the 1st day and started to increase SOD enzyme activity (P < 0.05). At the third day of the stress, SOD activities were changed 19.6 and 38.5% for X5671R and 5MX12956, respectively. At the 7th day, when the enzyme levels reached to the maximum level, these increase levels were determined as 42.9 and 69.4% respectively. As a result, SOD activities of both tomato varieties presented linear increase during the applied drought period. Similar results were found for sunflower (Zhang and Kirkham 1996), rice (Sharma and Dubey 2005), sesame (Fazeli et al. 2007), cucumber (Liu et al. 2009), wheat (Baisak et al. 1994) and many other crop plants.

Catalase enzyme catalysis increasing H_2_O_2_ levels to water and oxygen in plant cells produced as a side product of photoreactions (Luna et al. 2005). Catalase (CAT) enzyme activities are presented in Fig. 3D. Catalase enzyme activities of both varieties showed similar trendline as ascorbate peroxidase activities. Significant increase on X5671R enzyme activity observed from 2nd day, while 5MX12956 variety presented from the 3rd day of stress. At the 6th day of the stress when the highest CAT activity levels were observed for both varieties, 3.02- and 4.62-fold increase measured compared to Day 0 values of X5671R and 5MX12956 varieties, respectively. At the 7th day, catalase enzyme activities showed decrease in both varieties due to increasing oxidative damage. The measured CAT activity of X5671R was statistically significant at Day 7 and found 1.19-fold higher than Day 0, whereas no statistically significant activity change was determined between Day 0 and Day 7 for 5MX12956 variety (P < 0.05). Wang et al. (2009) applied drought stress by 35% PEG to six alfalfa tissue cultures for 7 days. They reported increased CAT enzyme activity on leaf samples of all six alfalfa varieties. Chugh et al. (2011) applied drought to 7 days old plantlets of six different maize varieties by withholding watering for 3 days. They reported increased CAT activity for two drought tolerant varieties and decreased CAT activities for 4 sensitive maize varieties. In our research, CAT activities of both varieties presented statistically significant increase for 6 days of drought (P < 0.05). However, in 7th day, plants could not tolerate the adverse effects of drought and CAT activities regressed to the Day 0 values. Similarly, Chakraborty and Pradhan (2012) applied drought stress to 5 different 30 days-old wheat varieties by withholding watering for 3, 6 and 9 days. They reported increased CAT activity at 3rd day for three varieties. After 3rd day, CAT activities of these same varieties decreased below control values suggesting that severity of stress is above the tolerance capacity of these varieties. Luna et al. (2005) applied drought stress to wheat by decreasing soil water content to 36–55% (mild drought) and 11–18% (severe drought). They reported significant CAT activity increase only in severe drought conditions.

#### Enzyme isozyme profiles

Isozymes are known to play a vital role in plant defense mechanisms (Johnson 2012). In this study, isozyme diversity of two industrial tomato varieties were investigated to identify band profiles as biochemical marker for the drought tolerance. Various crop plants also present different isozyme patterns during environmental stress conditions.

**Fig. 4:**
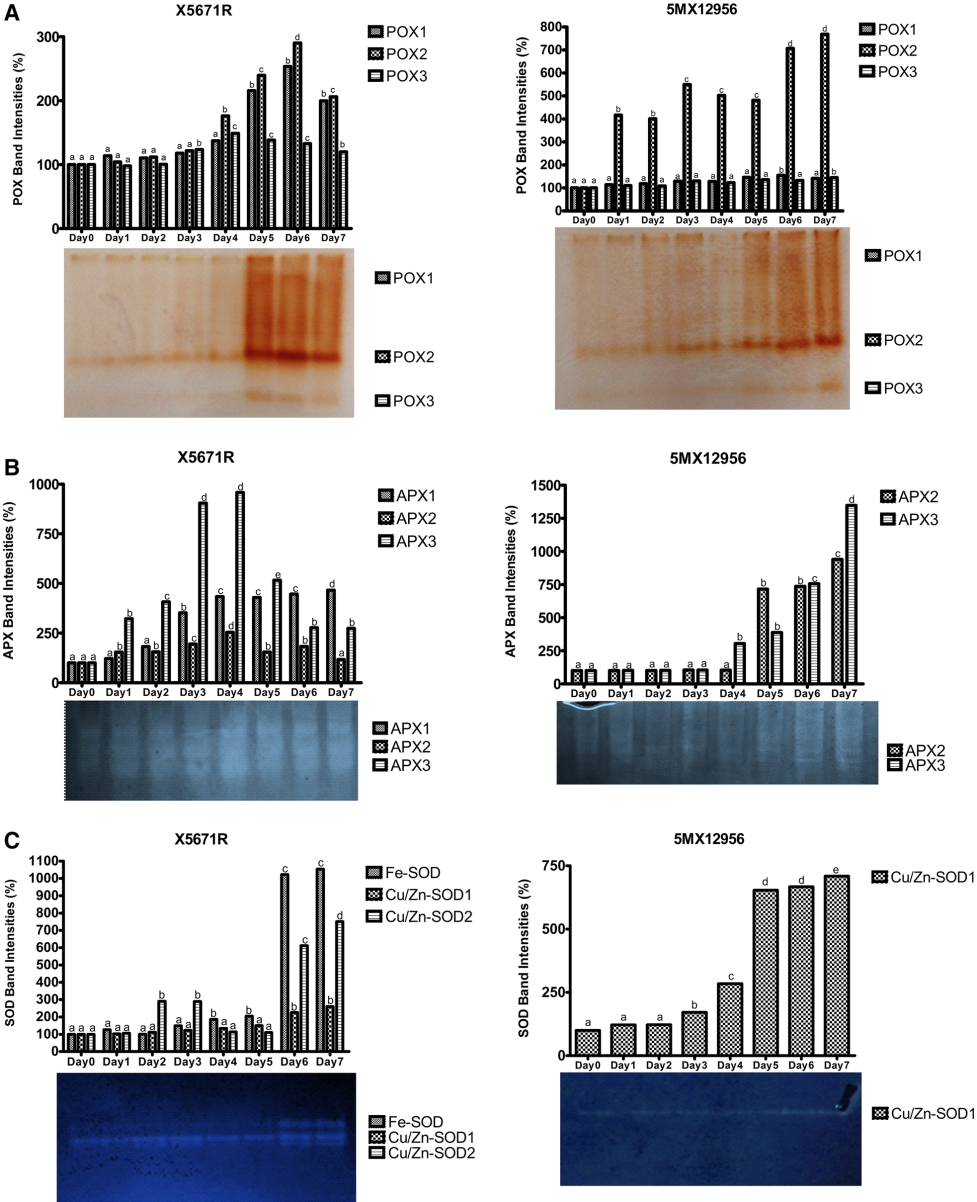
Effects of 7 day drought stress on the activity staining and % changes of POX (**A**), APX (**B**) and SOD (**C**) isozymes of X5671R and 5MX12956 tomato seedlings. Samples applied to the gels contained 50 µg protein for POX and APX and 75 µg for SOD. The *differentially given letters* represent significance at the 0.05 level

Peroxidase activity staining and band intensities belonging to the isozyme profiles of both varieties were presented on Fig. 4A, respectively. POX1, POX2 and POX3 isozymes were detected for both varieties. In this present study, POX isozyme activities of both tomato varieties increased due to the increasing drought especially after 4th day of treatment. All detected POX isozymes of X5671R variety increased for 6 days of drought treatment. After 6th day of treatment, expression levels of the isozymes were started to decrease in both varieties. POX1 and POX2 isozyme expressions reached to the maximum level at 6th day. However, at 7th day all isozyme band intensities showed decrease. Despite of this decrease all band intensities of Day 7 were higher than Day 0. The band intensities of POX1, POX2 and POX3 were 1.99-, 2.06- and 1.20-fold increased in comparison to the Day 0 values at 7th day of stress, respectively. However, POX isozyme band intensities were found higher than the values determined at Day 0 during the stress for X5671R variety. POX2 band intensity showed increasing pattern during the drought stress treatment in 5MX12956 variety. POX2 band intensity reached to the maximum level at 7th day of stress and was recorded as 7.68-fold higher than Day 0. According to the native PAGE results, increases in POX1 and POX2 isozymes are detected as the main reasons behind the increase of POX activity for X5671R variety, while only POX2 isozyme seems to effect the activity of POX enzyme for 5MX12956 variety.

Sen and Alikamanoglu (2014) applied drought to sugar beet tissue cultures by different concentrations of PEG6000. They detected eight POX isozymes after native PAGE separation. Among these eight isozymes, POX1 and POX5 were not detected in bands belonging to the control plants. Abedi and Pakniyat (2010) applied drought stress to 3 weeks old Brassica plantlets by reducing soil water capacity to 60 and 30%. They detected five POX isozyme bands after drought treatment. They detected increase in various POX isozymes suggesting the role of different POX isozymes to superoxide scavenging in response to drought conditions. Srivalli et al. (2003) applied drought stress to 50 days old rice plants by withholding water in 10, 13, 16 days periods. They detected four POX isozymes in response to drought treatment. They reported significant increase in POX3 isozyme which was higher than the control even after re-watering. POX2 and POX4 were not effected from application among the treatment but POX1 presented low abundance. They also indicated the importance of POX and SOD as key components against drought stress.

As another peroxide detoxifying enzyme APX activities of X5671R and 5MX12956 varieties presented via increase in isozyme pattern following the treatment days. Ascorbate peroxidase enzyme isozyme profiles of 7 days drought treated X5671R and 5MX12956 varieties were presented on Fig. 4B, respectively. APX1, APX2, APX3 isozymes were detected for X5671R variety, while 5MX12956 variety presented only APX2 and APX3 isozymes. For X5671R variety, band densities belonging to the APX1 isozyme increased at 7th day of the stress by 4.66-fold rate. While APX2 and APX3 band intensities reached the maximum levels at 4th day and then showed a decreasing profile until 7th day. Band intensity of APX3 isozyme showed a sharp increase rate by 3.22-fold on Day 1 and the intensity of this isozyme reached to 9.58-fold on 4th day of stress. At the 7th day of treatment APX2 and APX3 isozymes were determined higher than Day 0 by the 1.16- and 2.74-fold, respectively. Only APX2 and APX3 isozymes were detected for 5MX12956 variety after the activity staining of native polyacrylamide gels. Results are given in Fig. 4B. The band densities were increased in accordance with increasing drought intensity. We observed a sharp increase on Day 5 in APX2 isozyme by 7.16-fold, whereas APX3 isozyme showed 3.05-fold increase on Day 4. Although the band intensities showed similar increase rates by 7.36 and 7.56-fold at the 6th day of stress, responses of isozyme profiles presented differences on Day 7. APX2 band intensity reached to the maximum level by 9.4-fold while the density of APX3 isozyme was 13.48-fold at the 7th day of stress compared to Day 0. The variations in APX isozyme densities of both tomato varieties were found statistically significant (P < 0.05). Among detected APX1, APX2 and APX3 isozymes, APX1 and APX3 were more significant for X5671R variety. However, only APX2 and APX3 isozymes detected in 5MX12956 variety. X5671R isozymes were more intense comparing to the 5MX12956 variety. Their response profiles against drought stress were also different. In 5MX12956 variety, APX3 isozyme showed increase at 4th day initially; at 5th day, the increase rate of APX2 isozyme was higher than APX3 isozyme and on the 6th day, their activities were detected nearly the same. On the 7th day of the stress, APX3 isozyme density was observed 1.43-fold higher than APX2 isozyme. This isozyme difference between varieties also presented in accordance with APX activity results. Increase in the activities of APX in drought stressed plants are indicative of activation of ascorbate–glutathione cycle in tomato plants.

Şen and Alikamanoglu (2014) detected APX1 and APX2 in all samples while APX3 observed in most of the samples and APX4 was observed in all samples except the control. Srivalli et al. (2003) applied drought stress to 50 days old rice plants by withholding water in 10, 13, 16 days period. Six different APX isozymes were detected in native PAGE separation. APX2 isozyme was detected in all groups while APX3 and APX4 were only increased after drought treatment and decreased after re-watering. APX5 was effected negatively from drought and decreased after treatment. APX6 was found most dominant isozyme and kept uniform profile during the drought period. They suggested that differential appearance of APX isozymes may be result of differential functions of various APX isozymes in plants.

Another important element of antioxidative system is superoxide dismutase. SOD isozymes play their protective roles in different plant cell compartments. SOD isozyme band profiles showed differences between the tomato varieties as response to 7 day long drought stress. We detected Fe-SOD, Cu–Zn/SOD1 and Cu–Zn/SOD2 isozymes for X5671R variety, while only Cu–Zn/SOD1 isozyme was detected for 5MX12956 variety. For X5671R tomato variety, all SOD isozyme densities were increased during the stress treatment, but their increment rates were showed alterations among treatment days. Fe-SOD isozyme presented a sharp increase on Day 6 by 10.22-fold and continued to increase on Day 7. Cu–Zn/SOD1 showed gradual increase at 7th day of the stress and band density increased 2.60-fold compared to Day 0. The band density profiles of Cu–Zn SOD2 showed sharp increase at the 6th day. At the 7th day of the stress, activity of this enzyme was found as 7.51-fold increased with respect to Day 0. In 5MX12956 tomato variety, the only detected SOD isozyme was Cu–Zn/SOD1 and it showed increased pattern during the stress. After 5th day of the stress rapid increase was detected by 6.53-fold in comparison to Day 0. Last 3 days, the band intensities were increased severely by 7.09-fold, compared to Day 0 (P < 0.05). The changes in SOD isozyme activities were given in Fig. 4C.

Cu/Zn-SOD2 and Fe-SOD were determined as the most responsive isozymes against drought stress for X5671R variety. This result suggests the involvement of chloroplast to the early protection against oxidative stress caused by drought for this variety. However, in contrast with the X5671R variety, 5MX12956 variety presented only Cu/Zn-SOD1 isozyme increase while Cu/Zn-SOD2 and Fe-SOD isozymes were not detected. Fe-SOD is seem as the most responsive isozyme against drought in tolerant variety. Boaretto et al. (2014) applied drought stress by reducing relative water of soil in pot conditions to two different sugarcane cultivars. In water deficiency, they reported increased SOD enzyme activities for both varieties. They also reported two Mn-SOD and six Cu/Zn-SOD isozymes after drought treatment suggesting involvement of an extra SOD VI (Cu/Zn-SOD) to be involved in drought tolerance. Sekmen et al. (2014) applied combined drought and heat stress to two different cotton varieties. They reported one Mn-SOD, one Fe-SOD and two Cu/Zn-SOD isozymes for both varieties. They also suggested Cu–Zn/SOD2 to be the most responsive isozyme against drought in cotton. Abedi and Pakniyat (2010) applied drought stress to 3 weeks old Brassica plantlets by reducing soil water capacity to 60 and 30%. They detected two Mn-SOD isozymes and two Cu/Zn-SOD isozymes after stress application and suggested that the main reason in SOD activity increase may be related to Mn-SOD isozyme increase. Fe-SOD isozyme was not detected in this application. They discussed that Mn-SOD mainly function in mitochondrial electron transport chain and this cell compartment can be crucial for superoxide protection under stress.

## Conclusions

In conclusion, all the enzyme systems reveals the difference between the drought tolerant/sensitive varieties. The analysis results showed that the antioxidative system responses are different between two tomato varieties. The present study demonstrated specific changes in antioxidative system in two varieties of *S. lycopersicum*. The changes in isozyme patterns and enzyme activities were supported the sensitivity of 5MX12956 and tolerance of X5671R tomato varieties against 7 days long drought stress. The differences between antioxidative mechanism activities of two industrial tomato varieties that were initiated as a series of responses against drought stress has been proven via biochemical and electrophoretic analyses.

According to our results, increased POX1 and POX3 isozyme bands seem to have role in drought tolerance. Also, the main difference between the varieties is the absence of APX1 isozyme band in 5MX12956 tomato variety. Although there are differences in band intensities of APX2 and APX3 isozymes between the varieties during the stress treatment, the relation between the tolerance and APX1 isozyme should be evaluated. Another important isozymic difference was also observed between SOD banding patterns. Fe-SOD and Cu/Zn-SOD2 isozymes seem to be more effective in drought tolerance of X5671R tomato variety.

In further studies, the exact relations between tolerance isozyme differences need to be defined and their availability should be evaluated as potential biochemical markers for drought tolerance studies in breeding programs of these tomato varieties.
